# Supplementary material for: Use of motorised transport and pathways to childbirth care in health facilities: Evidence from the 2018 Nigeria Demographic and Health Survey
Source: PLOS Glob Public Health. 2022 Sep 21;2(9):e0000868. doi: 10.1371/journal.pgph.0000868 (PMC10021361; doi:10.1371/journal.pgph.0000868)
Supplement: S1 Table — (DOCX) [file pgph.0000868.s002.docx]

**S1 Table: Questions on transport and referral from section 4 (pregnancy and postnatal care) of the woman’s questionnaire (Source: 2018 NDHS)**

| **Number** | **Question** | **Answer choices (Last birth)** |
| --- | --- | --- |
| 430 | Where did you give birth?  *Probe to identify the type of source.*  *If unable to determine if public or private sector, write the name of the place.* | 1. Home    1. Her home   Skip to 434 if YES   - 1. Other home  1. Public Sector    1. Government hospital    2. Government health centre    3. Government health post    4. Other public sector (specify_______) 2. Private Medical Sector    1. Private hospital/clinic    2. Other private medical sector (specify________) 3. Other |
| 430A | Did you move from another health facility to come to this facility, or did you go directly from home to this facility, or from somewhere else that was not a health facility? | 1. Came from another health facility 2. Came from home   Skip to 430F   1. Came from other non-facility location 2. Don’t know |
| 430B | Which health facility referred or sent you to this facility where you gave birth?  *Probe to identify the type of source*  *If unable to determine if public or private sector, write the name of the place* | 1. Public Sector    1. Government hospital    2. Government health centre    3. Government health post    4. Other public sector (specify_______) 2. Private Medical Sector    1. Private hospital/clinic    2. Other private medical sector (specify________) 3. No formal referral 4. Other |
| 430E | What means of transportation did you use to get from the facility that referred you to the facility where you gave birth?  *Probe for the type(s) of transport used and record all mentioned* | 1. Motorised    1. Ambulance    2. Private car/truck    3. Taxi/Paid driver    4. Tricycle    5. Motorcycle/Scooter    6. Boat with motor    7. Public transport/bus 2. Not Motorised    1. Bicycle    2. Canoe/Boat without motor    3. Animal-drawn cart    4. Walking (On foot)    5. Carried 3. Other (Specify________) 4. Don’t know |
| 430F | What means of transportation did you use to get to the health facility where you gave birth?  *Probe for the type(s) of transport used and record all mentioned* | 1. Motorised    1. Ambulance    2. Private car/truck    3. Taxi/Paid driver    4. Tricycle    5. Motorcycle/Scooter    6. Boat with motor    7. Public transport/bus 2. Not Motorised    1. Bicycle    2. Canoe/Boat without motor    3. Animal-drawn cart    4. Walking (On foot)    5. Carried 3. Other (Specify________) 4. Don’t know |
