# Supplementary material for: Use of motorised transport and pathways to childbirth care in health facilities: Evidence from the 2018 Nigeria Demographic and Health Survey
Source: PLOS Glob Public Health. 2022 Sep 21;2(9):e0000868. doi: 10.1371/journal.pgph.0000868 (PMC10021361; doi:10.1371/journal.pgph.0000868)
Supplement: S2 Table — (DOCX) [file pgph.0000868.s003.docx]

**S2 Table: Proportions of motorised transport options used by women who gave birth in a health facility in the 2018 NDHS**

| **Among women who delivered in a health facility** | **Proportions of motorised transport options used** | | | | | | | | **Non-motorised transport** |
| --- | --- | --- | --- | --- | --- | --- | --- | --- | --- |
| **Variable** | **Total (N=9,015)** | **Ambulance (%)** | **Private car/truck (%)** | **Taxi (%)** | **Tricycle (%)** | **Motorcycle/Scooter (%)** | **Boat with motor (%)** | **Bus (%)** |  |
| **Total transport** |  | **6 (0.07)** | **1,783 (19.78)** | **1,377 (15.28)** | **532 (5.90)** | **2,754 (30.54)** | **35 (0.39)** | **362 (4.02)** | **2,166 (24.03)** |
| **Region of residence***** |  |  |  |  |  |  |  |  |  |
| North Central | 1,527 | 0 (0.00) | 202 (13.24) | 184 (12.04) | 31 (2.00) | 812 (53.17) | 15 (1.01) | 41 (2.66) | 242 (15.86) |
| North East | 1,031 | 3 (0.29) | 232 (22.48) | 127 (12.28) | 128 (12.41) | 341 (33.12) | 7 (0.70) | 21 (2.01) | 172 (16.71) |
| North West | 1,251 | 0 (0.00) | 522 (41.78) | 310 (24.79) | 76 (6.11) | 263 (21.00) | 0 (0.00) | 21 (1.72) | 58 (4.61) |
| South East | 1,728 | 0 (0.00) | 228 (13.18) | 169 (9.80) | 58 (3.33) | 532 (30.82) | 2 (0.12) | 42 (2.45) | 696 (40.30) |
| South South | 1,028 | 0 (0.00) | 155 (15.12) | 200 (19.51) | 65 (6.34) | 325 (31.60) | 5 (0.50) | 27 (2.64) | 250 (24.29) |
| South West | 2,452 | 3 (0.11) | 444 (18.11) | 387 (15.78) | 174 (7.10) | 481 (19.60) | 6 (0.23) | 210 (8.56) | 748 (30.51) |
| **Place of residence***** |  |  |  |  |  |  |  |  |  |
| Urban | 5,412 | 3 (0.06) | 1,287 (23.79) | 827 (15.29) | 445 (8.22) | 1,271 (23.48) | 16 (0.29) | 262 (4.85) | 1,300 (24.03) |
| Rural | 3,604 | 3 (0.08) | 496 (13.77) | 550 (15.26) | 87 (2.41) | 1,483 (41.15) | 19 (0.54) | 100 (2.77) | 866 (24.02) |
| **Wealth index***** |  |  |  |  |  |  |  |  |  |
| Lowest | 548 | 2 (0.31) | 44 (8.03) | 90 (16.47) | 9 (1.64) | 302 (55.07) | 1 (0.20) | 6 (1.10) | 94 (17.19) |
| Second | 1,109 | 0 (0.00) | 98 (8.83) | 149 (13.41) | 27 (2.41) | 523 (47.15) | 6 (0.56) | 33 (3.03) | 273 (24.61) |
| Middle | 1,856 | 1 (0.07) | 221 (11.90) | 247 (13.32) | 85 (4.60) | 736 (39.67) | 8 (0.42) | 57 (3.08) | 500 (26.92) |
| Fourth | 2,495 | 1 (0.06) | 392 (15.74) | 375 (15.03) | 197 (7.87) | 747 (29.94) | 6 (0.25) | 103 (4.11) | 674 (27.00) |
| Highest | 3,007 | 2 (0.05) | 1,028 (34.17) | 516 (17.16) | 214 (7.12) | 446 (14.82) | 14 (0.47) | 163 (5.41) | 625 (20.80) |
| **Referral***** |  |  |  |  |  |  |  |  |  |
| Came from home | 8,847 | 3 (0.03) | 1,729 (19.54) | 1,328 (15.01) | 519 (5.87) | 2,722 (30.77) | 32 (0.36) | 352 (3.98) | 2,162 (24.43) |
| Came from another health facility | 168 | 3 (1.97) | 54 (32.38) | 49 (29.16) | 13 (7.40) | 31 (18.49) | 3 (2.16) | 10 (5.92) | 4 (2.52) |
|  |  |  |  |  |  |  |  |  |  |
|  |  |  |  |  |  |  |  |  |  |
| **Among women who went to more than one facility** | **Total (N=168)** | **3** | **54** | **49** | **13** | **31** | **3** | **10** | **4** |
| **Initiation facility** |  |  |  |  |  |  |  |  |  |
| Government hospital | 30 | 0 (0.00) | 8 (27.95) | 7 (24.07) | 1 (2.61) | 7 (24.59) | 1 (4.63) | 5 (16.16) | 0 (0.00) |
| Government health centre | 73 | 3 (3.94) | 22 (30.41) | 26 (35.36) | 4 (5.74) | 11 (14.71) | 2 (3.08) | 1 (1.81) | 4 (4.95) |
| Government health post/other public sector | 21 | 0 (0.00) | 9 (42.48) | 5 (25.74) | 3 (10.20) | 3 (12.82) | 0 (0.00) | 2 (8.76) | 0 (0.00) |
| Private sector | 34 | 0 (0.00) | 12 (35.57) | 9 (25.63) | 2 (6.21) | 9 (26.31) | 0 (0.00) | 2 (5.76) | 0 (0.00) |
| No formal referral | 10 | 0 (0.00) | 3 (29.41) | 2 (20.10) | 3 (34.41) | 1 (15.99) | 0 (0.00) | 0 (0.00) | 0 (0.00) |

***: p-value <0.001
