# Supplementary material for: Use of motorised transport and pathways to childbirth care in health facilities: Evidence from the 2018 Nigeria Demographic and Health Survey
Source: PLOS Glob Public Health. 2022 Sep 21;2(9):e0000868. doi: 10.1371/journal.pgph.0000868 (PMC10021361; doi:10.1371/journal.pgph.0000868)
Supplement: S3 Table — (DOCX) [file pgph.0000868.s004.docx]

**S3 Table: Logistic regression model for use of motorised transport to place of childbirth as an outcome in the 2018 NDHS (N=21,823)**

| **Characteristics** | **Unadjusted model** | | | **Adjusted Wald test for variable** | **Adjusted model*** | | |
| --- | --- | --- | --- | --- | --- | --- | --- |
|  | **OR** | **95% CI** | **p-value** |  | **aOR** | **95% CI** | **p-value** |
| **Socio-demographic factors** |  |  |  |  |  |  |  |
| **Region of residence** |  |  |  | <0.001 |  |  |  |
| North Central | 2.56 | 2.11 – 3.12 | <0.001 |  | 1.68 | 1.37 – 2.07 | <0.001 |
| North-East | Ref |  |  |  | Ref |  |  |
| North-West | 0.64 | 0.52 – 0.80 | <0.001 |  | 0.62 | 0.52 – 0.74 | <0.001 |
| South-East | 3.40 | 2.73 – 4.23 | <0.001 |  | 0.73 | 0.57 – 0.93 | 0.011 |
| South-South | 2.20 | 1.77 – 2.73 | <0.001 |  | 0.55 | 0.43 – 0.70 | <0.001 |
| South-West | 3.96 | 3.22 – 4.87 | <0.001 |  | 0.72 | 0.56 – 0.93 | 0.010 |
| **Religion** |  |  |  | <0.001 |  |  |  |
| Christian | Ref |  |  |  | Ref |  |  |
| Islam | 0.33 | 0.29 – 0.37 | <0.001 |  | 0.85 | 0.72 – 1.00 | 0.057 |
| Traditional/Other | 0.23 | 0.12 – 0.46 | <0.001 |  | 0.68 | 0.38 – 1.19 | 0.174 |
| **Wealth index** |  |  |  | <0.001 |  |  |  |
| Lowest | Ref |  |  |  | Ref |  |  |
| Second | 1.96 | 1.65 – 2.32 | <0.001 |  | 1.38 | 1.17 – 1.62 | <0.001 |
| Middle | 4.14 | 3.45 – 4.96 | <0.001 |  | 1.88 | 1.57 – 2.26 | <0.001 |
| Fourth | 7.59 | 6.31 – 9.12 | <0.001 |  | 2.46 | 2.02 – 3.00 | <0.001 |
| Highest | 16.31 | 13.40 – 19.84 | <0.001 |  | 3.78 | 3.02 – 4.74 | <0.001 |
| **Highest education attained** |  |  |  | <0.001 |  |  |  |
| No education | Ref |  |  |  | Ref |  |  |
| Primary education | 2.89 | 2.52 – 3.32 | <0.001 |  | 1.36 | 1.19 – 1.56 | <0.001 |
| Secondary or higher | 7.41 | 6.54 – 8.41 | <0.001 |  | 1.88 | 1.63 – 2.16 | <0.001 |
| **Place of residence** |  |  |  | <0.001 |  |  |  |
| Urban | Ref |  |  |  | Ref |  |  |
| Rural | 0.29 | 0.26 – 0.33 | <0.001 |  | 0.80 | 0.70 – 0.91 | 0.001 |
| **Mother’s age at birth** |  |  |  | 0.048 |  |  |  |
| Less than 20 | 0.67 | 0.60 – 0.75 | <0.001 |  | 0.90 | 0.77 – 1.03 | 0.119 |
| 20 – 29 | Ref |  |  |  | Ref |  |  |
| 30 – 39 | 1.15 | 1.07 – 1.24 | <0.001 |  | 1.27 | 1.15 – 1.41 | <0.001 |
| 40 – 49 | 0.80 | 0.69 – 0.93 | 0.005 |  | 1.39 | 1.13 – 1.70 | 0.002 |
| **Pregnancy-related factors** |  |  |  |  |  |  |  |
| **Parity** |  |  |  | <0.001 |  |  |  |
| 1 | Ref |  |  |  | Ref |  |  |
| 2-3 | 0.84 | 0.76 – 0.93 | 0.001 |  | 0.76 | 0.67 – 0.87 | <0.001 |
| 4-5 | 0.58 | 0.52 – 0.64 | <0.001 |  | 0.58 | 0.49 – 0.68 | <0.001 |
| 6 or more | 0.36 | 0.33 – 0.40 | <0.001 |  | 0.55 | 0.46 – 0.66 | <0.001 |
| **Number of antenatal care visits** |  |  |  | <0.001 |  |  |  |
| None | Ref |  |  |  | Ref |  |  |
| 1-3 | 6.59 | 5.51 – 7.89 | <0.001 |  | 4.53 | 3.78 – 5.43 | <0.001 |
| 4-7 | 13.66 | 11.53 – 16.18 | <0.001 |  | 7.81 | 6.60 – 9.25 | <0.001 |
| 8 or more | 29.07 | 24.20 – 34.93 | <0.001 |  | 9.76 | 8.06 – 11.83 | <0.001 |
| **Complication woman might have experienced** |  |  |  |  |  |  |  |
| **Woman likely to have experienced at least one complication during labour or childbirth** |  |  |  | <0.001 |  |  |  |
| No | Ref |  |  |  | Ref |  |  |
| Yes | 3.69 | 3.21 – 4.25 | <0.001 |  | 3.01 | 2.55 – 3.55 | <0.001 |

Note: aOR= Adjusted Odds Ratio; CI= Confidence Interval; Ref=Reference Category; Adjusted Wald Test: This is used in STATA to test the goodness-of-fit of a model after adding an additional variable to the model in survey data.

*: Any variable within the model is adjusted for by all other variables within the same model
