# Supplementary material for: Use of motorised transport and pathways to childbirth care in health facilities: Evidence from the 2018 Nigeria Demographic and Health Survey
Source: PLOS Glob Public Health. 2022 Sep 21;2(9):e0000868. doi: 10.1371/journal.pgph.0000868 (PMC10021361; doi:10.1371/journal.pgph.0000868)
Supplement: S4 Table — (DOCX) [file pgph.0000868.s005.docx]

**S4 Table: Logistic regression model for use of motorised transport to place of childbirth as an outcome for all women whose most recent birth was in a health facility in the 2018 NDHS (N=8,927)**

| **Characteristics** | **Unadjusted model** | | | **Adjusted Wald test for variable** | **Adjusted model*** | | |
| --- | --- | --- | --- | --- | --- | --- | --- |
|  | **OR** | **95% CI** | **p-value** |  | **aOR** | **95% CI** | **p-value** |
| **Socio-demographic factors** |  |  |  |  |  |  |  |
| **Region of residence** |  |  |  | <0.001 |  |  |  |
| North Central | 0.99 | 0.65 – 1.50 | 0.954 |  | 0.89 | 0.56 – 1.39 | 0.597 |
| North East | Ref |  |  |  | Ref |  |  |
| North West | 3.91 | 2.34 – 6.55 | <0.001 |  | 2.61 | 1.60 – 4.26 | <0.001 |
| South East | 0.30 | 0.20 – 0.45 | <0.001 |  | 0.31 | 0.19 – 0.50 | <0.001 |
| South South | 0.59 | 0.38 – 0.91 | 0.018 |  | 0.50 | 0.30 – 0.84 | 0.008 |
| South West | 0.43 | 0.29 – 0.65 | <0.001 |  | 0.30 | 0.19 – 0.49 | <0.001 |
| **Religion** |  |  |  | 0.016 |  |  |  |
| Christian | Ref |  |  |  | Ref |  |  |
| Islam | 2.44 | 1.99 – 2.99 | <0.001 |  | 1.42 | 1.20 – 1.83 | 0.008 |
| Traditional/Other | 0.58 | 0.27 – 1.25 | 0.164 |  | 0.61 | 0.25 – 1.48 | 0.271 |
| **Wealth index** |  |  |  | <0.001 |  |  |  |
| Lowest | Ref |  |  |  | Ref |  |  |
| Second | 0.64 | 0.47 – 0.89 | 0.007 |  | 0.71 | 0.50 – 0.99 | 0.041 |
| Middle | 0.57 | 0.41 – 0.80 | 0.001 |  | 0.73 | 0.51 – 1.05 | 0.089 |
| Fourth | 0.58 | 0.41 – 0.81 | 0.001 |  | 0.82 | 0.57 – 1.18 | 0.297 |
| Highest | 0.83 | 0.59 – 1.19 | 0.314 |  | 1.18 | 0.79 – 1.76 | 0.426 |
| **Highest education attained** |  |  |  | 0.135 |  |  |  |
| No education | Ref |  |  |  | Ref |  |  |
| Primary education | 0.45 | 0.36 – 0.56 | <0.001 |  | 0.76 | 0.60 – 0.98 | 0.031 |
| Secondary or higher | 0.52 | 0.43 – 0.64 | <0.001 |  | 0.82 | 0.63 – 1.07 | 0.144 |
| **Place of residence** |  |  |  | 0.022 |  |  |  |
| Urban | Ref |  |  |  | Ref |  |  |
| Rural | 1.00 | 0.83 – 1.19 | 0.959 |  | 0.86 | 0.72 – 1.04 | 0.124 |
| **Mother’s age at birth** |  |  |  | 0.945 |  |  |  |
| Less than 20 | 1.27 | 1.02 – 1.58 | 0.034 |  | 0.95 | 0.74 – 1.21 | 0.662 |
| 20 – 29 | Ref |  |  |  | Ref |  |  |
| 30 – 39 | 0.95 | 0.83 – 1.07 | 0.385 |  | 1.12 | 0.96 – 1.31 | 0.165 |
| 40 – 49 | 0.99 | 0.77 – 1.29 | 0.964 |  | 1.17 | 0.87 – 1.58 | 0.290 |
| **Pregnancy-related factors** |  |  |  |  |  |  |  |
| **Parity** |  |  |  | <0.001 |  |  |  |
| 1 | Ref |  |  |  | Ref |  |  |
| 2-3 | 0.97 | 0.81 – 1.15 | 0.713 |  | 1.02 | 0.83 – 1.26 | 0.819 |
| 4-5 | 0.69 | 0.57 – 0.83 | <0.001 |  | 0.67 | 0.53 – 0.85 | 0.001 |
| 6 or more | 1.00 | 0.80 – 1.22 | 0.915 |  | 0.71 | 0.53 – 0.96 | 0.025 |
| **Number of antenatal care visits** |  |  |  | 0.002 |  |  |  |
| None | Ref |  |  |  | Ref |  |  |
| 1-3 | 0.73 | 0.49 – 1.08 | 0.112 |  | 0.66 | 0.45 – 0.98 | 0.049 |
| 4-7 | 1.00 | 0.71 – 1.43 | 0.980 |  | 1.08 | 0.77 – 1.52 | 0.652 |
| 8 or more | 0.74 | 0.52 – 1.04 | 0.085 |  | 1.16 | 0.81 – 1.66 | 0.425 |
| **Health service accessibility** |  |  |  |  |  |  |  |
| **Final facility of childbirth** |  |  |  | <0.001 |  |  |  |
| Government hospital | Ref |  |  |  | Ref |  |  |
| Government health centre | 0.24 | 0.19 – 0.30 | <0.001 |  | 0.35 | 0.28 – 0.44 | <0.001 |
| Government health post/other public sector | 0.21 | 0.14 – 0.33 | <0.001 |  | 0.23 | 0.14 – 0.37 | <0.001 |
| Private sector | 0.29 | 0.23 – 0.36 | <0.001 |  | 0.44 | 0.35 – 0.55 | <0.001 |
| **Referral** |  |  |  | 0.002 |  |  |  |
| Came from home | 1.00 |  |  |  | Ref |  |  |
| Came from another health facility | 12.00 | 2.77 – 51.95 | 0.001 |  | 8.87 | 1.90 – 41.40 | 0.006 |
| **Complication woman might have experienced** |  |  |  |  |  |  |  |
| **Woman likely to have experienced at least one complication during labour or childbirth** |  |  |  | <0.001 |  |  |  |
| No | Ref |  |  |  | Ref |  |  |
| Yes | 2.19 | 1.68 – 2.86 | <0.001 |  | 1.86 | 1.44 – 2.40 | <0.001 |

Note: aOR= Adjusted Odds Ratio; CI= Confidence Interval; Ref=Reference Category; Adjusted Wald Test: This is used in STATA to test the goodness-of-fit of a model after adding an additional variable to the model in survey data.

*: Any variable within the model is adjusted for by all other variables within the same mode
