# Supplementary material for: Use of motorised transport and pathways to childbirth care in health facilities: Evidence from the 2018 Nigeria Demographic and Health Survey
Source: PLOS Glob Public Health. 2022 Sep 21;2(9):e0000868. doi: 10.1371/journal.pgph.0000868 (PMC10021361; doi:10.1371/journal.pgph.0000868)
Supplement: S5 Table — (DOCX) [file pgph.0000868.s006.docx]

**S5 Table: Proportion of women who used motorised transport to final facility of childbirth among women who were referred (N=168) in the 2018 NDHS**

| **Characteristics** | **Total sample (N=168)** | **Proportion of women who used motorised transport to place of childbirth N (%)** | ***p*-value (could not be determined for all due to small sample)** |
| --- | --- | --- | --- |
| **Socio-demographic factors** |  |  |  |
| **Mother’s age at birth** |  |  |  |
| Less than 20 | 15 | 15 (100.0) |  |
| 20 – 29 | 82 | 78 (95.0) |  |
| 30 – 39 | 63 | 63 (99.7) |  |
| 40 – 49 | 8 | 8 (100.0) |  |
| **Highest education attained** |  |  |  |
| No education | 35 | 35 (100.0) |  |
| Primary education | 21 | 21 (100.0) |  |
| Secondary or higher | 112 | 108 (96.2) |  |
| **Religion** |  |  |  |
| Christian | 112 | 108 (96.6) |  |
| Islam | 55 | 55 (100.0) |  |
| Traditional/Other | 1 | 1 (100.0) |  |
| **Wealth index** |  |  |  |
| Lowest | 12 | 12 (100.0) |  |
| Second | 22 | 22 (100.0) |  |
| Middle | 30 | 30 (100.0) |  |
| Fourth | 49 | 45 (92.6) |  |
| Highest | 55 | 54 (98.9) |  |
| **Marital status** |  |  |  |
| Never in union | 2 | 2 (100.0) |  |
| Currently in union | 154 | 151 (97.6) |  |
| Formerly in union | 12 | 11 (95.1) |  |
| **Place of residence** |  |  |  |
| Urban | 105 | 101 (96.4) |  |
| Rural | 63 | 63 (100.0) |  |
| **Region of residence** |  |  |  |
| North Central | 18 | 17 (96.6) |  |
| North East | 20 | 20 (100.0) |  |
| North West | 29 | 29 (100.0) |  |
| South East | 26 | 26 (100.0) |  |
| South South | 26 | 24 (88.6) |  |
| South West | 49 | 48 (98.8)) |  |
| **Pregnancy-related factors** |  |  |  |
| **Parity** |  |  |  |
| 1 | 51 | 51 (100.0) |  |
| 2-3 | 59 | 56 (94.9) |  |
| 4-5 | 30 | 29 (99.4) |  |
| 6 or more | 28 | 28 (100.0) |  |
| **Number of antenatal care visits** |  |  |  |
| None | 5 | 5 (100.0) |  |
| 1-3 | 23 | 23 (100.0) |  |
| 4-7 | 75 | 74 (99.2) |  |
| 8 or more | 65 | 61 (94.4) |  |
| **Location of antenatal care** |  |  |  |
| None | 5 | 5 (100.0) |  |
| Home | 3 | 3 (100.0) |  |
| Government hospital | 43 | 43 (100.0) |  |
| Government health centre | 74 | 70 (95.1) |  |
| Government health post/other public sector | 10 | 10 (100.0) |  |
| Private medical sector | 33 | 33 (100.0) |  |
| **Complication woman might have experienced** |  |  |  |
| **Woman likely to have experienced at least one complication during labour or childbirth** |  |  |  |
| No | 72 | 71 (98.6) |  |
| Yes | 96 | 93 (96.6) |  |
|  |  |  |  |
| **Health service accessibility** |  |  |  |
| **Initial facility woman accessed for childbirth care** |  |  |  |
| Government hospital | 19 | 18 (97.7) |  |
| Government health centre | 49 | 48 (98.8) |  |
| Government health post/other public sector | 18 | 18 (100.0) |  |
| Private sector | 82 | 79 (96.1) |  |
| **Final facility of childbirth** |  |  |  |
| Government hospital | 79 | 78 (98.7) |  |
| Government health centre | 14 | 14 (100.0) |  |
| Private sector | 75 | 71 (95.7) |  |
