# Supplementary material for: Use of motorised transport and pathways to childbirth care in health facilities: Evidence from the 2018 Nigeria Demographic and Health Survey
Source: PLOS Glob Public Health. 2022 Sep 21;2(9):e0000868. doi: 10.1371/journal.pgph.0000868 (PMC10021361; doi:10.1371/journal.pgph.0000868)
Supplement: S6 Table — (DOCX) [file pgph.0000868.s007.docx]

**S6 Table: Breakdown of numbers for use of motorised transport represented in the Sankey diagram (Figure 1; n=168)**

| **Facility of referral origin** | **Facility of final childbirth** | **Number of women who used motorised transport** |
| --- | --- | --- |
| Government hospital | Government hospital | 18 |
|  | Government health centre | 1 |
|  | Government health post/other | 0 |
|  | Private sector | 10 |
| Government health centre | Government hospital | 41 |
|  | Government health centre | 7 |
|  | Government health post/other | 0 |
|  | Private sector | 26 |
| Government health post/other | Government hospital | 13 |
|  | Government health centre | 6 |
|  | Government health post/other | 0 |
|  | Private sector | 3 |
| Private sector | Government hospital | 7 |
|  | Government health centre | 1 |
|  | Government health post/other | 0 |
|  | Private sector | 35 |
