# Supplementary material for: Use of motorised transport and pathways to childbirth care in health facilities: Evidence from the 2018 Nigeria Demographic and Health Survey
Source: PLOS Glob Public Health. 2022 Sep 21;2(9):e0000868. doi: 10.1371/journal.pgph.0000868 (PMC10021361; doi:10.1371/journal.pgph.0000868)
Supplement: Attachment — Submitted filename: Response to Reviewers.docx [file pgph.0000868.s010.docx]

Ghana College of Physicians and Surgeons

No. 54 Independence Avenue, Ridge

Accra, Ghana.

12^th^ July 2022

Dickson Abanimi Amugsi, PhD

Academic Editor

PLOS Global Public Health

Dear Editor,

Thank you very much for considering our paper and for the reviewer’s comments. We have addressed these comments as set out in the table below while ensuring that we stayed within the word limit for the original research paper.

We have also taken the opportunity to read through the entire manuscript again to improve the language where we deem necessary. We have uploaded an unmarked version as well as a track changes version of the revised manuscript for your review.

Thank you for your consideration and we look forward to your response.

Best wishes,

Dr Cephas Ke-on Avoka

on behalf of all authors

|  | **Comments** | **Response** |
| --- | --- | --- |
|  | **Editor’s comments** |  |
| 1 | Thank you for submitting your manuscript to PGPH for publication. Three independent reviewers have reviewed your manuscript. Although they felt it has merit, they raised substantial issues that will need your attention | Thank you. We have made efforts to address all the issues raised by all reviewers within the manuscript. We have also attempted to enlighten the reviewers on the methodology of our research and the rationale behind our analysis and conclusions. |
| 2 | There is lack of clarity in many places. There is also a need to take a second look at the data analysis, especially the issue regarding the suitability of analysing motorised transport use in non-facility births | Thank you. We have addressed these comments from the reviewers directly and explained further in the manuscript. |
| 3 | It is important to revisit the limitations of the study. The discussion section needs to be situated within the study findings, and not opinions that are not established by study | We thank the editor and reviewer for this suggestion and have revised the discussion section accordingly. We have grounded our study findings in the relevant literature to aid in the interpretation our results. We offer some hypotheses about why we see the results we do but that were not possible to explore fully given the limitations of the data available. We present these as areas for further exploration in this under-researched topic. |
| 4 | Lines 137-138: How did you arrive at this decision | Thank you for your comment. We have decided to exclude the records of the 88 women missing information on where they came from to give birth and the means of transport used. This now reads in our manuscript as: “Among women who delivered in a health facility, 88 women (1% of all facility deliveries) were missing information on where they came from and the means of transport to facility of birth. We excluded these records in our facility-level, bivariate and logistic regression analysis” |
| 5 | Why did you present both unadjusted and adjusted ORs, when the interpretation is based on the adjusted ORs. It makes the tables unnecessarily busy. I will suggest you take out the unadjusted ORs | We thank the editor for this suggestion. We now present only the adjusted ORs in the main manuscript. We added a supplementary table with the unadjusted ORs for the interest of readers. |
|  |  |  |
|  | **Reviewer 1** |  |
| 1 | This is really important and need to make understandable on what and how to use such transport mechanisms in the far health facilities. I was interested if the study includes the proportion of the motors, vehicles or ambulances availability and its utilization by mothers. If you can include the availability of motors in this study, that can help the readers to understand transport utilization in health system | Thank you for the comment. The data collected in the Nigeria Demographic and Health Survey (NDHS) included a specific list of motorised and non-motorised transport options. Women who were interviewed were asked to select from this list, the means of transport used to get to the health facility and for referral in the intrapartum. We replicated this part of the questionnaire in supplementary table 1 (S1 Table), questions 430E and 430F for reference. We therefore conducted our analysis based on this information.  We attempted to provide additional information on the proportion of women who used each of the motorised transport options in the questionnaire. This can be found in supplementary table 2 (S2 Table).  We are unable to provide or explain the availability of each of these motorised transport options for women as we do not have that data in the NDHS. We do however indicate that the most common mode of transport used by women who deliver in health facilities in Nigeria is the motorcycle. This reads as: “The data from Uganda showed a higher prevalence of motorised transport to reach a facility for childbirth (91% compared to 76% in our study), but the commonest mode of transport by women who delivered in a health facility in Uganda and in our study in Nigeria was motorcycle taxis.” |
| 2 | You put the recommendations – Obstetric transport interventions should be affordable, adequate and bridge the equity gap in utilisation of these services; but I couldn’t get any result in that aspect | Thank you for the comment. We have now clarified this in the manuscript. This reads as: “obstetric transport interventions should address the unmet need in access and utilization of life-saving services by pregnant women” |
| 3 | Analyzing motorized transport use in non-facility births is not good; better to remove this part. The first thing – they may want to use and not accessible – no information about that; the second thing – it make confusion for the readers – different results in one article | Thank you for your comment. Our study includes both women who deliver at home and women who deliver in a health facility. Given that less than half of all women in Nigeria deliver in a health facility, analysing use of motorised transport in facility births only would greatly underestimate the proportion of women using motorised transport to their place of childbirth in the country. The addition of non-facility births to our analysis therefore only increases the denominator for the sample of all women. We explained this in our paper which reads as: “To enable us to determine the use of motorised transport in the total sample of respondents in the survey, women who gave birth at home or in other non-facility locations were added to those who used non-motorised transport but were excluded when only women who gave birth in a health facility were assessed “  Additionally, we hypothesised that the types of transport used would differ if all women in Nigeria were compared to only women who deliver in a health facility; and our study findings indeed proved this hypothesis to be right. This finding reads in our paper as: “However, when we examined motorised transport use among only women who gave birth in health facilities, different patterns of use and potential need became apparent.”  We have included a statement in the methods to provide a clearer rationale for the structure of our analysis. This reads as: “We hypothesised that the characteristics, patterns, and determinants of use of transport to childbirth would differ between all women in Nigeria and only women who deliver in a health facility, and thus conducted our analysis comparing these two categories of women”. This analysis is shown in Table 2. |
| 4 | It is understandable that when you use secondary data you can draw multiple limitations, so better to minimize the limitations to increase the study creditability | Thank you. We have now stated only the most salient limitations in our study. |
| 5 | Your study objective was specifically on motorized transport use in non-facility births, but you assessed and presented several issues including place of birth, referring places and its determinants. So, it is better to consistent across the document including study objective, results, discussion, conclusion, and recommendations | Thank you for your comment. We wish to clarify that our study objective was to describe transport pathways and use of motorised transport for all women in Nigeria. This reads in our study as: “Our study aims to describe transport pathways used by all women who give birth in health facilities in Nigeria (including pathways for women who are referred) and to assess the determinants of the use of motorised transport to health facilities for childbirth care using the 2018 NDHS.”  We therefore structured our analysis to explore the transport pathways and differences in proportions of women (all women in Nigeria and women who go to health facilities for childbirth) who used motorised transport based on socio-demographic and pregnancy-related factors, and to highlight the determinants of use of motorised transport, comparing all women in Nigeria to women who gave birth in a health facility. |
|  |  |  |
|  | **Reviewer 2** |  |
| 1 | The study is based on a questionnaire employing personal recall from the previous 5 years. The subjects filling out the questionnaire could mis-remember, mis-understand the questions, or be in a hurry and fill out the questionnaire hastily, either misstating or omitting information. Thus, the personal recall questionnaire is notoriously prone to error  In addition, five years is too long to have accurate recall and there was no method of substantiating or corroborating the recalled answers. I would have considered the preceding 3 years as reasonably reliable and the preceding 1 year as highly reliable  Finally, the period over five years could include substantial changes in societal capacities and behaviours that affect the results  It is unclear how rates of literacy affected the results of this study but seems clear that one cannot fill out a questionnaire without reading/writing skills, every country has a finite illiteracy, and yet there was no uncertainty added for this probability or any discussion of its likelihood. For those who were illiterate, was assistance filling the form provided or permitted? Or were all forms filled out by a second person asking the questions? | Thank you for your comment. Our study is based on data from the 2018 Nigeria Demographic and Health Survey (NDHS). The DHS Program is funded by the United States Agency for International Development (USAID) which offers financial support and technical assistance for population and health surveys in several countries worldwide.  The 2018 NDHS used computer-assisted personal interviewing (CAPI) for data collection. A pre-test training was designed for trainers on the NDHS to ensure they were well versed with the NDHS questionnaire and procedures and able to test the questionnaires in different languages. The training focused on key components of the survey, interview techniques and procedures for completing the NDHS questionnaire and administration of interviews. Training of field staff was facilitated by the ICF team and participants were evaluated through class exercises and observations made during field practice. More information on data collection for the DHS can be found through the reference below:  “The DHS Program - Nigeria (2018) The DHS Program - Nigeria: DHS, 2018 - Final Report (English). <https://dhsprogram.com/publications/publication-fr359-dhs-final-reports.cfm>. “  Indeed, we considered the five-year recall period to potentially introduce some bias in the responses. We captured this in our limitation which reads: “..the data were also subject to potential errors with recall and reporting on transport and referral pathways, given that women were asked these questions (recalling their last pregnancy in the five years preceding the survey) with little to no means of verification.”  Additionally, we attempted to mitigate this limitation by restricting our analysis to the most recent live birth in the five-year period. This reads in our methods as: “The unit of analysis was restricted to the most recent live birth per woman within the previous five years”  The woman’s questionnaire of the DHS, from which we conducted our analysis, is designed to collect data from every woman interviewed, on their “last birth” and “next-to-last birth” in the last five years preceding the survey. It is therefore not feasible to restrict our analysis to a recall period less than what was captured in the dataset.  We have included a statement in the paper to provide more information on data collection. This reads as: “The 2018 NDHS used computer-assisted personal interviewing (CAPI) for data collection by highly trained enumerators” |
| 2 | For those 88 questionnaires with missing information, they should not have been included. Instead, some information was inferred for the missing data, but they were still included in the study. Inferring results when not known is inappropriate. Although the results might not have tipped the balance to a different outcome, it seems there were enough responses with complete information that the 88 responses were not required to reach statistically reliable conclusions | Thank you for your comment. We have now excluded the records of these women from our facility-level, bivariate and logistic regression analysis. This however, did not change our results or conclusions in anyway. We have modified the methods to read: “Among women who delivered in a health facility, 88 women (1% of all facility deliveries) were missing information on where they came from and the means of transport to facility of birth. We excluded these records in our facility-level, bivariate and logistic regression analysis.” |
| 3 | The study is a cross-sectional study which is not the best type of study as (for one thing) there is no control group included. The study design makes drawing definitive conclusions problematic  Some of the results could easily be attributed to reverse-causation. That should be pointed out | Thank you for your comment. Indeed, we appreciate the limitations associated with cross-sectional studies and do not in any way draw any definitive causal relationships in our analysis and interpretation. We limited our discussion to drawing relevant logical relationships between our variables and the descriptive patterns we observed in our study. |
| 4 | There were some results that seemed trivial or obvious. For instance, among women transferred from another health facility 98% used motorized transport. But it would seem obvious that transfer from one facility to another is a result of an obstetrical complication, and it would not be possible to use a non-motorized transport during a health emergency. How useful is this result? How useful is the study if it only tells one what must be so? If the statistics confirm for us women living in more affluent areas, have higher education, or come from the most affluent quintile are more likely to use motorized transport, is that a surprise? Is it valuable information? If so, how may it be used? If it is not ‘new’ information, the reader’s interest is not captured and the journal impact is lessened | Thank you for your comment. Our study was informed by the limited availability of large-scale data to understand pathways and mode of transport to childbirth care. It is for this reason that we set out to initially describe these patterns before exploring any determinants of use of motorised transport. What is described as obvious by the reviewer could only be considered an assumption, rather than the facts that our study provides. We highlighted this rationale in our study which reads as: “..there is limited availability of large-scale data such as the periodically reported Demographic and Health Surveys in LMICs to understand pathways and mode of transport to care. Where these data exist, there are large gaps in key components of referral, mainly because this information is not captured routinely.”  Additionally, it is only by comparing the sample of all women in Nigeria to only women who deliver in a health facility that the different patterns of education and socio-economic status are exposed, which is again novel information that our study contributes to the literature on travel of pregnant women for childbirth. |
| 5 | It would have been useful if the questionnaire had asked about motivations for using or not using motorized transport to obtain delivery care. As the authors pointed out, the facts alone do not reveal whether women intended to use motorized transport or not and how their plans for transportation worked or did not work at the time they gave birth. For instance, the following statement was made:  “Understanding travel to reach health facilities and factors that influence transport and referral mechanisms is a critical step needed to develop an effective obstetric transport and referral system [12].”  Does this study reveal the factors that influence the transport that was used? It is unclear. | Thank you for your comment. As stated in the response to an earlier comment, the DHS employs a standard woman’s questionnaire to collect data from the sample of women interviewed. There are considerable limitations in adding more questions to an already lengthy DHS questionnaire. We have therefore highlighted the need for further studies to explore the motivations and intentions of women who use motorised transport for childbirth. This reads in our paper as: “Further studies are needed to understand where women intend to deliver, how experiencing pregnancy or childbirth complications might influence this decision and the use of different motorised transport options.”  Some studies have been conducted to explore issues of maternity care-seeking and referral (references below), but these have not been done to our knowledge, at a nationally representative level.   1. Banke-Thomas A, Balogun M, Wright O, Ajayi B, Abejirinde IOO, Olaniran A, et al. Reaching health facilities in situations of emergency: qualitative study capturing experiences of pregnant women in Africa’s largest megacity. Reproductive Health [Internet]. 2020 Sep 25 [cited 2022 Apr 6];17(1):145. Available from: <https://doi.org/10.1186/s12978-020-00996-7> 2. Wong KLM, Brady OJ, Campbell OMR, Banke-Thomas A, Benova L. Too poor or too far? Partitioning the variability of hospital-based childbirth by poverty and travel time in Kenya, Malawi, Nigeria and Tanzania. International Journal for Equity in Health [Internet]. 2020 Jan 28 [cited 2022 Mar 30];19(1):15. Available from: <https://doi.org/10.1186/s12939-020-1123-y> |
| 6 | Because the data were expressed as odds ratios, they were expressed as relative to some data categorical result, arbitrarily set to 1. Since the choice of reference is arbitrary, this would necessarily affect the results. Should be pointed out and discussed. | Thank you for your comment. The choice of reference category in reporting odds ratios is not arbitrary. This is informed by other studies that have used similar categories of variables, the sample size of each category, the ease of interpretation, as well as the conceptual framework of our study. One category in each variable has to be set as a reference (comparator), which is commonly noted as “ref” or “1” in tables reporting results. Either way, this does not change the conclusions that we draw from our analysis since the odds ratios are always reported and explained taking the reference category into consideration. |
| 7 | At the end of reading the manuscript, my question was how can these data be used? The statement above was that understanding travel to reach health facilities is a critical step to develop effective obstetric transport. How and where do these results fit into fulfilling that goal? The audience is missing critical statements that would connect the dots between the important research questions and the stated goals of better obstetrical transport pathways. Not sure that the data collected, and their analysis was able to answer the questions. | Thank you for your comment. To the best of our knowledge, our study is the first to provide nationally representative estimates of use of motorised transport by pregnant women for childbirth, thanks to the contributions made by some of our study authors (ER, OMRC, LB) in adapting the woman’s questionnaire of the 2018 NDHS. Our study further highlights the patterns underlying the use of motorised transport at the national level. As mentioned in our study, Nigeria is currently in the process of establishing a national ambulance service. Our study therefore provides important insight to inform the design of this service, especially for women seeking childbirth care. For instance, our study indicates that women who experience complications of childbirth and women who are referred are more likely to use motorised transport. Policy makers could therefore make a case for prioritising these groups of women in the design of the national ambulance service. We have highlighted this in our study which reads as: “Nigeria is currently in the process of establishing a national ambulance service [29]. However, such services need to be delivered on a large-scale and set up closer to communities”  Additionally, our study highlights important gaps in understanding transport pathways especially for women with stillbirths and post-partum complications. Capturing such data on nationally representative surveys such as the DHS will further strengthen interventions to improve obstetric transport pathways. |
| 8 | Restriction to live births information, and from living mothers who gave birth, is going to skew the data because the worst outcomes, the unthinkably bad outcomes, are those where transport affects the viability of mother or offspring, yet these were not collected. I feel this does skew the data toward the less severe outcomes and muddles the truth. The authors touch on this in their last section of the discussion. | Thank you for your comment. We have highlighted this in our limitations. This reads as: “The NDHS asked the childbirth care-seeking questions used in this analysis only of women with a live birth. There is evidence that many women needing emergency obstetric care experience stillbirth (and some women die), but these women and the pathways and modes of transport they use, if any, are not captured in this dataset”  Other studies have attempted to provide this additional information (as seen in the reference below), but not at a nationally representative level. Our study therefore provides a means to validate the DHS as potential source of providing this information at scale.   1. Banke-Thomas A, Avoka CK on, Gwacham-Anisiobi U, Omololu O, Balogun M, Wright K, et al. Travel of pregnant women in emergency situations to hospital and maternal mortality in Lagos, Nigeria: a retrospective cohort study. BMJ Global Health [Internet]. 2022 Apr 1 [cited 2022 Jun 30];7(4):e008604. Available from: <https://gh.bmj.com/content/7/4/e008604> |
| 9 | The statement was made that “Obstetric transport interventions should be affordable, adequate and bridge the equity gap in utilisation of these services.” However, this study did not measure the affordability or adequacy in utilization of motorized services. Affordability would be measured by responses to questions about the cost of transport, but here there were none. Adequacy would be measured by responses to questions about the plans for transport before birth and the availability of choices, together with the actual option used and justification for using that option, or alternatively would include information about the total number of motorized vehicles available for hire or of public or private transport capacity of the system at the time of birth. But these questions were not part of the study. Therefore, the statement has nothing to do with the study results. It is an opinion or judgment and is not a supported conclusion from these data. | Thank you for your comment. We have now modified this in the manuscript to read as: “obstetric transport interventions should address the unmet need in access and utilization of life-saving services by pregnant women”.  Given that our study highlights the important socio-economic variations in use of motorised transport among all women in Nigeria, we believe future interventions should be geared towards addressing these variations. |
| 10 | Although the STROBE checklist was provided, not all categories contained a response | Thank you for your comment. This has now been completed accordingly. |
| 11 | The Discussion section contains quite a bit of opinion that seems to be designed to support policy decisions. I don’t really have a problem with any of the statements, they seem well-intentioned. However, the authors should be cautioned that the Discussion should be directly tied to the data presented in the manuscript and not depart far from the factual conclusions that can be directly reached using these data. That is overreaching | Thank you for your comment. To the best of our ability, we have discussed the results of our study in the context of Nigeria, and in relation to relevant literature. We believe we have grounded our findings the literature and proposed hypothesis for further research where relevant. An example of this in our study reads as: “As per our findings (and from the NDHS), only 41% of women give birth in health facilities in Nigeria. This is strongly patterned by region. Wong and colleagues have shown that the predicted probability of hospital birth in Nigeria was low for those with either a long travel time or from poorer households” |
| 12 | Uses a national survey which one would assume to be carefully organized and conducted. It is a well-written manuscript. The statistical methods employed appear valid and adequate. Organization of the results and their presentation was good. | Thank you for your comments. Indeed, the integrity of the DHS as a source of nationally representative data on salient demographic and health indicators cannot be overemphasised. A quote from the 2018 DHS report for Nigeria (referenced below) reads as: “The information collected through the 2018 NDHS is intended to assist policymakers and programme managers in evaluating and designing programmes and strategies for improving the health of the country’s population”  The DHS Program - Nigeria (2018) The DHS Program - Nigeria: DHS, 2018 - Final Report (English). <https://dhsprogram.com/publications/publication-fr359-dhs-final-reports.cfm>. |
| 13 | Certain of the conclusions and observations are very salient, e.g., “Our study showed that women who delivered in a health facility and used motorised transport to get there had nine times the odds of being referred from one facility to another (compared to those who came from home) and three times the odds of reporting at least one possible complication during labour or childbirth (multiple gestation, caesarean section or an early neonatal death) compared to women who delivered at home.” | Thank you for your comments. To the best of our knowledge, our study is the first to make these salient conclusions using a nationally representative survey. |
| 14 | For Table 1 (Sociodemographic, obstetric factors and transport pathways….) it is not clear to me which comparison the p-value is referring to, as it is listed on the first line in each variable. For instance, ‘mother’s age at birth’ has a p-value for the ‘less than 20’ line entry of <0.001 just to the right of the block titled “women’s most recent live birth <5 years before the 2018 NDHS (n=21,911), so I think this value applies to the comparison of non-facility births vs. facility births among <20 years old, but what about the groups 20-29, 30-39, 40-49? There is no p-value listed beside these lines, what does that mean? Does it mean the comparison wasn’t made, wasn’t significant, or some other conclusion? The remainder of the data table is laid out the same way, with only a p-value in the first line of the variable | Thank you for your comment. P-values in Table 1 are reported for the whole variable, and not the categories within the variable. The P-value therefore provides evidence of the difference in column estimates reported for each category of the variable. We have improved the clarity of the tables overall. |
| 15 | The same question re Table 2. There is no explanation in the text for the way the p-value is presented, and it is not intuitive. On page 14, line 180-181, the statement is made that “(Table 2, column B, p<0.001 for all)”. Then, if the p-value is the same for each comparison in the group and for all groups, it should be stated in the table somewhere. Otherwise, one cannot tell even if all the comparisons were run. And if it is true the p-value is the same for every comparison within the group, for all groups (all are presented the same way), it seems a little strange. Statistical comparisons are rarely that neat and tidy. | Thank you for your comments. P-values in Table 2 are also reported for the whole variable, and not the categories of the variable. The P-values therefore provide evidence for the difference in proportions of the use of motorised transport (row percentages) in each category of the variable. The statement “p<0.001 for all” is just an indication that there is strong evidence for a difference in proportions of use of motorised transport among all the categories of variables reported (for the sample of all women in Nigeria).  We have improved the clarity of the tables overall. |
| 16 | Table 3. For each categorical variable, one choice was chosen as the index and assigned a value of “1”. Was there a basis for this choice? What was it? As mentioned above, the presentation of the results is biased by the choice of index. | Thank you for your comment. As mentioned in an earlier response to the comments, the selection of the reference category is informed by literature and the conceptual framework of the study. Results of odds ratios are always reported with regards to the reference category chosen for the variable.  The results of the study therefore are not biased by the choice of the index, as in all studies reporting odds ratios, the reference categories are always taken into consideration in reporting of results. |
| 17 | Table 3 – The column “Adjusted Wald test for variable” needs further explanation in the footnote or within the text. It would be easier for the reader if this is included in the footnote to the table, to save having to hunt for it in the text. What is the value of performing this adjustment and what happens to the result when it is done? It this adjustment critical or of value? Based on a cursory comparison of the unadjusted and adjusted Wald test columns, there is not a difference in the result, and inclusion does not therefore seem to be of value. It just adds more columns to the table without providing further information. You could add a note in the text or footnote that the Wald test was performed and yielded the same results. | Thank you for your comment. We have now included a note on the Adjusted Wald Test as a footnote on the Tables 3 and 4. The Adjusted Wald Test is meant to test the goodness-of-fit of the model after the addition of a variable to the model and should not be interpreted in relation to unadjusted or adjusted models. The reported odds ratios and corresponding p-values are the only indices to be compared.  We have also improved the clarity of the tables overall. |
| 18 | S4 Table – n= 168 the total number of observations adds to 165. S3 Table – please check the math, not all categories sum to 168 | Thank you for your comments. We have corrected the figures in the relevant tables and improved the clarity of the tables overall. The numbers in Table S4 do add up to 168. |
|  | The data and conclusions can stand alone, however, I suggest that the authors may want to collect further information that would make the manuscript more interesting and informative. | Thank you for your comment. Our analysis is limited by the data provided by the DHS. We have however recommended areas of further studies to improve our understanding of the subject under study. This reads as “Our study exposes several potential areas of future research focus to further our understanding of travel, transport and referral patterns of women seeking childbirth care” |
|  |  |  |
|  | **Reviewer 3** |  |
|  | The formatting of the article needs to be improved as prescribed by the journal guidelines while language and punctuation improvements are required in the introduction and findings. | Thank you for your comment. We have reviewed the article and improved the language and punctuations. |
|  | The line 73/74 would be better written with punctuation as”, Despite the recognition of critical research needs; to better understand how women access essential care at child birth while referral and transport systems remain poorly documented and researched”. | Thank you for your comment. We believe the punctuation (comma) is ideally situated to differentiate the recognition of the need for further research and the current poorly documented/researched status. |
|  | In line 81 author mentioned the adaptation of the standard Women’s Questionnaire for country priorities. Please mention for what purpose was it adapted and what is the relevance of this particular evidence in current study? Please make the clear mention. | Thank you for your comment. We highlighted in our rationale the need for large-scale data to understand pathways to childbirth care. This reads as: “Thus far, there is limited availability of large-scale data such as the periodically reported Demographic and Health Surveys in LMICs to understand pathways and mode of transport to care. Where these data exist, there are large gaps in key components of referral, mainly because this information is not captured routinely”  The questionnaire was thus adapted to allow the DHS to capture such data routinely. We have clarified this statement in the paper which now reads: “To improve data collection and better understand pathways and mode of transport to care, the standard Woman’s Questionnaire of the 2018 Nigeria Demographic and Health Survey (NDHS) was adapted for country priorities”  Some of our authors have highlighted gaps in the coverage of key components of referral in the study referenced below.   1. Radovich E, Banke-Thomas A, Campbell OMR, Ezeanochie M, Gwacham-Anisiobi U, Ande ABA, et al. Critical comparative analysis of data sources toward understanding referral during pregnancy and childbirth: three perspectives from Nigeria. BMC Health Services Research [Internet]. 2021 Sep 6 [cited 2022 Mar 18];21(1):927. Available from: <https://doi.org/10.1186/s12913-021-06945-9> |
|  | The methodology has been very much rigorously written while I would like the author to clearly mention the rationale/logic behind recoding the missing values as such. | Thank you for your comment. We have now excluded missing values from our facility-level analyses. This reads in our methods as: “Among women who delivered in a health facility, 88 women (1% of all facility deliveries) were missing information on where they came from and the means of transport to facility of birth. We excluded these records in our facility-level, bivariate and logistic regression analysis” |
|  | Punctuation missing in the line no 168 could better be rephrased for clear reading. | Thank you for your comment. The phrase now reads: “Most of the women who were subsequently referred, delivered in either a government hospital or private facility (right-hand side of Sankey diagram).” |
|  | The presentation of the findings is very fine and I hope it meets up the journal requirements. The findings could be discussed with more other relevant articles in context of developing and developed countries as well. I hope authors could find relevant publications as well regarding the pathway of delivery. | Thank you for your comment. We appreciate the value you place on the findings and the suitability of the manuscript for this journal.  We deemed it prudent to discuss the findings in a developing country context and highlighted its relevance to existing literature on pathways to childbirth care in Nigeria and other countries in sub-Saharan Africa. |
